# Draft Genome Sequence of Exopolysaccharide-Producing Thermophilic Bacterium *Brevibacillus thermoruber* Strain 423

**DOI:** 10.1128/genomeA.00774-13

**Published:** 2013-09-26

**Authors:** Songul Yasar Yildiz, Margarita Kambourova, Kazim Yalcin Arga, Ebru Toksoy Oner

**Affiliations:** Department of Bioengineering, Marmara University, Goztepe, Istanbul, Turkeya; Department of Extremophilic Bacteria, Institute of Microbiology, BAS, Sofia, Bulgariab

## Abstract

*Brevibacillus thermoruber* strain 423 is a Gram-positive, spore-forming, aerobic, and thermophilic bacterium that produces mannogalactoglucan exopolysaccharide (EPS). We report the draft genome sequence of *B. thermoruber* 423, which will accelerate research on the cellular organization of thermophilic bacteria, as well as the rational design and optimization of EPS production.

## GENOME ANNOUNCEMENT

The last decade witnessed significant improvements in the discovery and development of new microbial exopolysaccharides (EPSs) from extremophiles that possess novel industrial significance (1). Among them, thermophiles constitute the least explored group, with very limited literature on them despite their fast metabolism enabling high production rates (2). Given the need for a good EPS-producing thermophile that could be used as a model organism to study the biological mechanisms and whole-genome organization of EPS-producing thermophilic bacteria, *Brevibacillus thermoruber* strain 423, isolated from a hot spring (temperature, 59°C; pH 6.5) in southwest Bulgaria and found to excrete high titers of EPS outperforming reported yields (3), was sequenced via next-generation sequencing (NGS) technology. *B. thermoruber* has been identified as a Gram-positive, motile, red-pigmented, aerobic, and thermophilic bacterium (4).

In this study, whole-genome sequencing of *B. thermoruber* 423 was performed via Illumina HiSeq 2000 technology. Paired-end sequencing was conducted with two different insert sizes of 500 and 2,000 bp. Filtering of the raw data (669 Mb) for low-quality bases, adapter contamination, and duplicates resulted in clean sequence data (603 Mb) consisting of 958,150 reads (mean length of 454 bp) and 95,666 reads (mean length of 2,446 bp), providing approximately 150-fold coverage. The reads were assembled via the SOAP*denovo* software version 1.05 (5), using a *k*-mer size of 15 bp and the key parameter K setting at 71. The assembly process resulted in 15 scaffolds, with a total length of 4.43 Mbp, a maximum length of 783,797 bp, and an N_50_ value of 596,854 bp. BLAST (6) alignments of the assembled scaffolds indicated statistically significant genomic similarity to other *Brevibacillus* species. The RAST server version 4.0 (7) was used to predict and annotate the genes of the draft genome sequence. The SEED viewer version 2.0 (8) was used to categorize predicted genes into functional subsystems.

The resultant draft genome sequence had a mean G+C content of 58.46%, 4,446 coding sequences, and 112 RNA genes. Of the coding sequences, 42% were assigned to subsystems. Putative functions were assigned to 3,020 protein-coding genes, whereas 1,426 hypothetical proteins had no match to any known proteins. Phylogenetic analysis of the draft genome sequence revealed a rather close relationship to *B. thermoruber* strain JAM-FM2501, with 99% similarity based on 16S rRNA sequence (GenBank accession no. KF192950) comparison. However, whole-genome comparison with the genome sequences available at the RAST server indicated closest similarity to *Brevibacillus brevis* NBRC 100599 (score, 500).

The whole-genome sequencing of *B. thermoruber* 423 marks an important step toward the rational genetic and metabolic optimization of EPS production. Furthermore, the information provided by the whole-genome sequence will accelerate research on EPS production from thermophilic microorganisms via elucidation of the biological mechanisms related to EPS production, and it serves as a framework for metabolic reconstructions of thermophilic microorganisms and as an analysis platform for the study of microbial polymer production.

### Nucleotide sequence accession numbers.

This whole-genome shotgun project has been deposited in DDBJ/EMBL/GenBank under the accession no. ATNE00000000. The version described in this paper is the first version, ATNE01000000.
